# COVID-19 research priorities for non-pharmaceutical public health and social measures

**DOI:** 10.1017/S0950268821000716

**Published:** 2021-04-05

**Authors:** Jan C. Semenza, Cornelia Adlhoch, Agoritsa Baka, Eeva Broberg, Orlando Cenciarelli, Stefania De Angelis, Margot Einoder-Moreno, Irina Jovel Quinonez Dalmau, Pete Kinross, John Kinsman, Katrin Leitmeyer, Angeliki Melidou, Howard Needham, Diamantis Plachouras, Emmanuel Robesyn, Senia Rosales-Klintz, Jonathan E. Suk, Carl Suetens, Klaus Weist, Andrea Würz, Pasi Penttinen

**Affiliations:** European Centre for Disease Prevention and Control (ECDC), Gustav III:s boulevard 40, 169 73 Solna, Sweden

**Keywords:** COVID-19

## Abstract

Europe is in the midst of a COVID-19 epidemic and a number of non-pharmaceutical public health and social measures have been implemented, in order to contain the transmission of severe acute respiratory syndrome coronavirus 2. These measures are fundamental elements of the public health approach to controlling transmission but have proven not to be sufficiently effective. Therefore, the European Centre for Disease Prevention and Control has conducted an assessment of research gaps that can help inform policy decisions regarding the COVID-19 response. We have identified research gaps in the area of non-pharmaceutical measures, physical distancing, contact tracing, transmission, communication, mental health, seasonality and environment/climate, surveillance and behavioural aspects of COVID-19. This prioritisation exercise is a step towards the global efforts of developing a coherent research road map in coping with the current epidemic but also developing preparedness measures for the next unexpected epidemic.

Non-pharmaceutical public health measures are currently playing a very important role in reducing the spread of COVID-19 [1]. Some of these measures may to a lesser extent be evidence based [2]. Further research may inform the current or future policies. In this assessment, we outline a number of research gaps that can help inform policy decisions regarding the COVID-19 response.

The European Centre for Disease Prevention and Control (ECDC) has commented on gaps in knowledge and research in a number of technical guidance documents [3, 4]. Other bodies have drawn attention to lack of good quality evidence on topics including: the level of distance for physical distancing, the effectiveness of masks when used in the community, the effectiveness of various measures in daily life settings such as transport, work, public services of all kinds, tourism; vehicle and building design, air conditioning, surface cleaning techniques, the effectiveness of different personal protective equipment (PPE) in health and social care; other techniques to protect health care workers and evaluations of different systems for isolation and contact tracing.

The World Health Organization (WHO) has developed a research and development (R&D) Blueprint in order to rapidly expand scientific knowledge on severe acute respiratory syndrome coronavirus 2 (SARS-CoV-2), to track its spread and virulence and to provide advice to countries and individuals on control measures [5]. Key knowledge gaps and research priorities and shared scientific data on ongoing research were identified, thereby accelerating the generation of critical scientific information to contribute to control of the COVID-19 emergency. The R&D Blueprint research priorities include activities to interrupt transmission immediately and to prepare for future and immediate research actions to be considered [5]. The research gaps for non-pharmaceutical public health and social measures identified by the WHO are highlighted in the WHO R&D Blueprint strategy and the 2019 Novel Coronavirus Global Research and Innovation Forum [5, 6]. The Global research collaboration for infectious disease preparedness (GloPID-R) network brings together 28 leading research funders for the purpose of facilitating an effective research response for (re-) emerging infectious disease threats. It has worked on ensuring research synergies for COVID-19 that are identified across geographical areas and disciplines [7].

The European roadmap to lifting coronavirus containment measures describes how to gradually lift the non-pharmaceutical public health and social measures, and how to enter the recovery phase and revitalising society and the economy. Three criteria to be fulfilled before the non-pharmaceutical public health and social measures should be lifted, have been defined. They include epidemiological criteria, health system capacity and monitoring capacity to quickly detect and isolate infected individuals. To do so, a number of steps need to be taken and include:

- Gather data and develop a robust system for reporting. Harmonised gathering and sharing of data at national and subnational levels by public health authorities is essential to better manage the lifting of measures.
- Create a framework for contact tracing and alerting with the possible use of mobile apps, while respecting data privacy.
- Testing capacities must be expanded and harmonised. Fast and reliable testing is key to swift diagnoses and to measure the population's acquired immunity. The Commission has presented guidelines on coronavirus tests.
- The capacity and resilience of health care systems should be increased. Particularly, to address the predicted rise in infections after rolling back the containment measures. The EU budget has been mobilised to help.
- The availability of medical and PPE should be improved. The Commission supports Member States by stockpiling and distributing supplies and equipment via rescEU and Joint Procurement.
- Develop and fast-track the introduction and distribution of vaccines, treatments and medicines. The deployment of safe and effective vaccines against SARS-CoV-2 and variants of concern would be essential in putting an end to the coronavirus outbreak.

In order to refine the specific COVID-19 research priorities for non-pharmaceutical public health and social measures we conducted an assessment with the ECDC Public Health Emergency (PHE) experts working on the response to the COVID-19 pandemic.

The current state of COVID-19 science and the existing knowledge gaps were assessed through daily automatic searches of the peer-reviewed literature in PubMed with the following search string: ‘COVID-19’[Supplementary Concept] OR ‘severe acute respiratory syndrome coronavirus 2’[Supplementary Concept] OR ‘COVID-19 vaccine’[Supplementary Concept] OR ‘COVID-19 serotherapy’[Supplementary Concept] OR ‘COVID-19 diagnostic testing’[Supplementary Concept] OR ‘COVID-19 drug treatment’[Supplementary Concept] OR ‘LAMP assay’[Supplementary Concept]* OR ‘Coronavirus Infections’[Mesh:noexp] OR ‘Wuhan coronavirus’[TW] OR ‘Wuhan seafood market pneumonia virus’[TW] OR COVID19[TW] OR ‘COVID-19’[TW] OR ‘COVID-2019’[TW] OR ‘coronavirus disease 2019’[TW] OR ‘SARS-CoV-2’[TW] OR SARS2[TW] OR ‘2019-nCoV’[TW] OR ‘2019 novel coronavirus’[TW] OR ‘severe acute respiratory syndrome coronavirus 2’[TW] OR ‘2019 novel coronavirus infection’[TW] OR ‘coronavirus disease 2019’[TW] OR ‘coronavirus disease-19’[TW] OR ‘novel coronavirus’[TW] OR coronavirus[TW] OR ‘SARS-CoV-19’[TW] OR ‘SARS-CoV-2019’[TW].

The retrieved publications were collated in an Endnote database and subjected to a review of title and abstract by two investigators (IJQD and SRK). Relevant publications of high quality and impact were compiled into a weekly digest and distributed to ECDC PHE experts working on infection prevention and control (IPC), clinical aspects of COVID-19, experts in microbiology, preparedness, response, surveillance, scientific evidence and modelling. Moreover, specific literature searches in these disciplines were conducted to complement the general search on COVID-19 science. New scientific breakthroughs in the field of COVID-19 epidemiology were presented to ECDC technical experts at a daily roundtable and technical group meetings at ECDC in order to continuously update ECDC PHE technical experts about the latest developments in the field and research gaps.

Based on the technical expertise of these ECDC PHE experts and based on requests from the European Commission and EU Member States the research gaps for non-pharmaceutical public health and social measures were assessed using a two-phased approach: first, ECDC PHE experts were consulted about research areas which will require an expanded evidence base according to their technical assessment. Relevant information on this topic was used as a reference point [5, 6, 8]. These experts were asked to identify research gaps in their area of expertise.

In a second round, these ECDC PHE experts were requested to prioritise the research gaps for non-pharmaceutical public health and social measures based on the public health impact and importance. Disagreements regarding these priorities were resolved during PHE expert team meetings. This document was shared with the Health Security Committee of the European Commission for feedback from the different member states.

In light of the WHO R&D Blueprint and the European roadmap [5, 8], knowledge gaps and uncertainties reduce the confidence in policy implementation to optimise the public health response. Therefore, there is a need to detail some of these persistent knowledge gaps into research action, and prioritise research to create knowledge that best serves the most pressing policy needs. The list of priorities included in Table 1 represents a selection of research priorities as seen from a scientific expert perspective. They include research gaps in the area of non-pharmaceutical measures, physical distancing, contact tracing, transmission, communication, mental health, seasonality and environment/climate, surveillance and behavioural aspects of COVID-19.

**Table 1.** Selected research priorities for non-pharmaceutical public health and social measures according to ECDC experts

ECDC research priority	Why is this a priority?	What types of studies/research are needed?
*Non-pharmaceutical measures*		
Quantify the contribution of different non-pharmaceutical public health and social measures such as travel restrictions and physical distancing measures on the trajectory of the epidemic	Prioritise interventions according to impact	Multivariate modelling of high-resolution COVID-19 incidence data stratified by age, sex and NUTS3 location
Differentiate and validate various COVID-19 response strategies including ‘intermittent physical distancing’, ‘open society’, ‘universal mass testing’, ‘lock down’ and ‘segmenting and shielding’	Gradual opening of society by allowing certain low risk activities	Multivariate modelling of response measures
*Physical distancing*		
Assess effectiveness, impact and compliance of particular non-pharmaceutical public health and social measures by analysing changes in population mobility, social contact and associated disease patterns	Prioritise interventions	Information on patterns of travel is used extensively by software companies, often via apps that users have permitted to track their location, for example, so that public transport users can use an app to discover when the next bus is due. These data can now be used to assess the effectiveness of physical distancing measures
How to improve societal resilience to non-pharmaceutical public health and social measures in order for countries to cope, adapt and transform under these strict measures	Enhance effectiveness of interventions	Focus groups, polls and structured interviews
*Contact tracing*		
Assess pattern of transmission of COVID-19 and effectiveness of outbreak control through contact tracing mobile applications	Identification of contacts and contact tracing for containment and treatment	Analysis of anonymised smartphone data from tracing apps and epidemiological modelling
Understand social acceptance of mobile applications for contact tracing and their associated ethical/data protection dimensions	Overcome barriers to widespread use of contact tracing apps Policy development for handling of confidential data (centralised *vs*. decentralised)	Focus groups, polls and interviews
Assess the efficacy of contact tracing and its impact on preventing transmission	Operationalise contact tracing	Collect and analyse available data on: proportion of new cases arising from known contacts; number of contacts per case (by exposure category, by age of case and contacts, over time); proportion of contacts reached out; proportion of contacts actively/passively followed for quarantine period
*Transmission*		
The role of children in transmission and the implication for school closures	Inform need for, timing and duration of school closures	Evaluate mitigation measures in schools in relation to outbreaks in schools, infection rates or sero-prevalence rates. Due to children being often asymptomatic, needs wide sampling policies
Enhanced understanding of underlying mechanics of virus dissemination from both a/pre symptomatic individuals, and clinically ill	Key parameter which will inform a large number of public health measures, research and modelling, as well as enhance confidence in basic social distancing advice (e.g. 1–2 m)	Replicate droplet and aerosol dispersal in various natural settings, including possible animal models to inform actual transmission risk. Detailed epidemiological investigations of cases (e.g. super-spreaders) and outbreaks in a variety of settings, particularly in high incidence situations
Transmission in primary care, acute care, long-term care facilities both to patients/residents and healthcare workers in relation to settings and activities as well as applied IPC measures (standard care, intensive care, aerosol-generating procedures, collection of diagnostic respiratory samples, social contact with colleagues)	Determine the relative role of different settings, activities and interventions in transmission and the relative protective effect of various components of PPE (medical face masks, respirators, gowns, eye protection – goggles and visors, gloves)	Cohort and case-control studies in hospitalised patients and healthcare workers. Environmental testing studies. Healthcare-associated and occupational infection outbreak investigations
The relative role of fomites, large respiratory droplets, aerosols and faecal oral transmission in the COVID-19 pandemic	Important to target preventive measures to the most common routes of transmission and ensure effective bundles of measures	Environmental testing studies (PCR and viral culture) Experimental studies of transfer of the virus from surfaces to hands Studies of survival of the virus on the skin Development of models of infection Outbreak investigations
The role of face masks in the community in order to clarify under which use face masks are warranted	Determine if face masks in the community should be endorsed	Epidemiologic and experimental investigations of the efficacy of face masks Research on materials for reusable-washable face masks
Transmission in public transportation vehicles (buses, trains, airplanes and other transport)	Minimise transmission risk while offering opportunity for passenger travel; support transport industries to recommence activities	Transmission dynamics in public vehicles and optimising mitigation options (vehicle disinfection, passenger management, outbreak investigations of airline passengers, environmental testing)
Effectiveness of different disinfection approaches	Improved hygiene by optimising cleaning and disinfection practises in different settings to reduce transmission	Create new disinfectants; for example one that is heat activated at 37 °C. Detailed, setting specific, studies to assess vial load and potential infectivity following application of different cleaning and disinfection protocols. Create a disinfectant that is heat activated at 37 °C
*Communication*		
Implications of inconsistent and/or changing public health advice for public understanding, trust and adherence to the recommended/mandated measures	Advance the uptake of public health measures	Focus groups, surveys, interviews
Analysis of non-pharmaceutical public health and social measures (including behavioural interventions, nudging etc.) to identify good practices	Refine the communication approach to reach different segments of society	Focus groups, surveys, interviews
Understand and combat dis/misinformation, stigma and fear about COVID-19 in social media	Intercept and prevent dis/misinformation, stigma and fear about COVID-19 in social media	Content analysis of social media outlets
*Mental health*		
Psychological aspects due to social isolation and lock down, including risk of anxiety, depression and other outcomes, such as self-harm and suicide	Avoid unintended consequences of interventions	There might be an increased risk of depression and other mental health conditions due to the increased risk of limited access to lifesaving treatments for acute medical conditions, and due to the increased risk of suboptimal clinical management or screening for chronic medical conditions
Domestic violence, and maternal and neonatal health aspects due to social isolation and lock down	Device intervention measures for prevention	Quantify these impacts in epidemiologic studies
*Seasonality and environment/climate*		
The transmission dynamic of SARS-CoV-2 depends on a number of factors, including the timing and extent of control measures, duration of host immunity to SARS-CoV-2, cross-immunity between SARS-CoV-2 and other human coronaviruses and the strength of seasonal forcing on transmission	Will seasonality supress the epidemic in the summer months?	Analytic studies (e.g. simulation models), to determine whether climatic factors, such as temperature, humidity or UV, will suffice to supress the transmissibility of SARS-CoV-2 during the summer months in the Northern Hemisphere
Examine the association between incidence/mortality rates due to COVID-19 and air pollutants	Enact air pollution reduction measures	Model the contribution of air pollutants such as PM_10_, NO*x*, etc. on the epidemiology of COVID-19
*Surveillance*		
RNA detection and quantification of SARS-CoV-2 in wastewater	Monitor the effectiveness of non-pharmaceutical public health and social measures	Screen large populations and communities in order to detect outbreaks early (early warning system: prior to individual-level diagnosis) and track the outbreak in real-time
Evolution of seroprevalence in the community and among specific risk groups	Provides information on the cumulative exposure. Support monitoring the effectiveness of non-pharmaceutical public health and social measures	Population-based (or risk-group or setting based) seroprevalence studies performed on a regular basis or longitudinally
Monitoring of prevalence of infection in the community and among specific risk groups	Provides information on the current transmission levels. Support monitoring the effectiveness of non-pharmaceutical public health and social measures	Population-based (or risk-group based) PCR prevalence studies performed on a regular basis or longitudinally
Comprehensive population-based surveillance of severe infection	Provides more comparable data on the incidence of severe COVID-19 disease in populations and on outcomes.	Population-based surveillance of severe acute respiratory infections with testing for SARS-CoV-2, influenza and other respiratory pathogens
*Behavioural*		
Changes in risk perception, behaviours and measure fatigue due to long-term implementation of non-pharmaceutical public health and social measures	Counteract changes in risk perception	Examine longitudinal changes in awareness and behaviour change in relation to public risk communication

The selection of research gaps below includes the most relevant areas for public health and those which may have a direct impact on the modification or adaptation of measures. These research priorities are outlined to complement the European roadmap to lifting COVID-19 containment measures [8].

The compilation of this overview of research gaps is based on the daily review of the global epidemiologic situation by the ECDC epidemic intelligence team. Moreover, it is also based on a review of the thematic areas that are covered by the PHE team at ECDC.

As Europe is facing a resurgence of the pandemic following relaxation of social distancing measures, the need for understanding the effectiveness of countermeasures that are applicable and appropriate for a variety of settings is urgent. A number of important research gaps remain that need to be addressed in order to contain the transmission of COVID-19. Of particular importance is the need to better understand the mechanism of transmission of COVID-19 and which non-pharmaceutical measures are the most effective at mitigating the epidemic.

These research gaps can only be achieved by collectively respecting the framework for successful research partnerships in global health [9]. This outline of research priorities for non-pharmaceutical public health and social measures was posted on the EU Health Policy Platform under COVID-19 research to policy action in order to help EU research consortia prioritise their activities. It was validated against the COVID-19 Research Project Tracker by UKCDR and GloPID-R, which is a live database of funded research projects across the world related to the current COVID-19 pandemic, as part of the COVID CIRCLE initiative [10]. Of 661 funded epidemiological research projects at the time of writing, 198 projects (30%) pertained specifically to mitigation measures including non-pharmaceutical interventions on transmissibility. In comparison, 384 funded research projects (58%) addressed transmission dynamics including pre-symptomatic/asymptomatic transmission, 154 (23%) disease severity including the role of different age groups in transmission and 58 (9%) susceptibility including the infectivity of children (overlapping categories). Our assessment did also capture these additional aspects (Table 1) in light of non-pharmaceutical public health and social measures.

These global research efforts confirm the persistence of knowledge gaps regarding non-pharmaceutical public health and social measures. International collaborations between teams and transparency in this process will reduce duplication of work and provide a clearer picture of the epidemiology of COVID-19. It can help accelerate and optimise the implementation of non-pharmaceutical public health and social measures to help contain the spread of SARS-CoV-2. It can also provide a strategic approach to counter the infodemic of mis/disinformation associated with the COVID-19 pandemic, which requires swift, regular, systematic and coordinated action from multiple sectors of society and government [11]. WHO developed research questions to prioritise the practice of infodemic management [12]. They were organised into five streams to assure focus, structure and a methodology that's rooted in evidence: (1) measuring and monitoring the impact of infodemics during health emergencies; (2) detecting and understanding the spread and impact of infodemics; (3) responding and deploying interventions that protect against the infodemic and mitigate its harmful effects; (4) evaluating infodemic interventions and strengthening resilience of individuals and communities to infodemics and (5) promoting the development, adaptation and application of tools for managing infodemics.

This prioritisation exercise for research on non-pharmaceutical public health and social measures is a step towards the global efforts of developing a coherent research road map [5] in coping with the current epidemic but also developing preparedness measures for the next unexpected epidemic.

## Conflict of interest

None.

## Data availability statement

The data that support the findings of this study are openly available at: https://www.ecdc.europa.eu/en.
